# Investigating the Effect of the 10° Reverse Trendelenburg Position on Spinal Block Characteristics and Hemodynamic Parameters in Lower Limb Surgeries

**DOI:** 10.7759/cureus.22588

**Published:** 2022-02-25

**Authors:** Mahesh Kumar, Shyam Bhandari, Aman Thakur, Sunil Thakur, Ravinder Verma, Bhanu Awasthi

**Affiliations:** 1 Anesthesiology, Civil Hospital, Thural, IND; 2 Anesthesiology, Dr. Rajendra Prasad Government Medical College and Hospital, Kangra, IND; 3 Anesthesiology, Dr. Radhakrishnan Government Medical College, Hamirpur, IND; 4 Anesthesiology and Critical Care, Dr. Rajendra Prasad Government Medical College and Hospital, Kangra, IND; 5 Orthopedics and Trauma, Dr. Rajendra Prasad Government Medical College and Hospital, Kangra, IND

**Keywords:** spinal anesthesia, torso, trendelenburg position, local anesthetic, bradycardia, hypotension

## Abstract

Introduction: The primary goal of spinal anesthesia in lower limb surgeries is to achieve a successful sensory and motor block. Adequate level of spinal block for lower limb orthopedic surgery is T10. Due to multiple factors affecting the level of spinal anesthesia, it is not always easy to control the level of spinal anesthesia.We proposed that maintaining patients in a 10° reverse Trendelenburg position after spinal anesthesia can significantly control the height of the sensory block, resulting in stable hemodynamics.

Materials and methods: This study is a single centric, prospective, single-blinded randomized clinical trial (CTRI/2018/08/015455) conducted in a tertiary care center in Sub-Himalayan region in India from July 2018 to June 2019. Total 60 patients fulfilling our inclusion and exclusion criteria were recruited in the study and were divided into two groups. In the supine group, patients were positioned in the supine position, and in the Trendelenburg group, patients were positioned in a 10° reverse Trendelenburg position after administering spinal anesthesia with 12.5 mg bupivacaine heavy. The two groups were compared in terms of sensory block, motor block, and analgesia duration. Heart rate, blood pressure, mean arterial pressure, and hypotension were also compared between the two groups.

Results: Duration of sensory block, motor block, and analgesia were significantly higher in patients of the reverse Trendelenburg group (group T) compared to the supine group (group S). In group T, 26.6% had a sensory block level above T8, whereas in group S, 86.6% of patients had a sensory block level above T8. No hypotension was observed in the Trendelenburg group, which was present in 33% of patients in the supine group (group S).

Conclusion: Ten-degree reverse Trendelenburg position immediately after giving spinal anesthesia significantly limits the level of sensory block and provides better hemodynamic stability, and can be more beneficial, especially in geriatric patients and other high-risk patients for lower limb surgeries.

## Introduction

The primary goal of spinal anesthesia in lower limb surgeries is to achieve a successful sensory and motor block. Several factors including the age of patients, height and weight, local anesthetics, positioning of the patient being administered the anesthetic drug, dosage, injection technique, patient-specific symptoms, and needle level orientation are known to influence the level and duration of sensory block [1,2]. Because of the compromised cardiopulmonary reserve, a high prevalence of hypotension and bradycardia has been reported in the case of spinal anesthesia, which could be risky, especially in the elderly age group [3].

The spread of local anesthetics once injected into the cerebrospinal fluid is determined by many factors, the most important of which is patient positioning. During spinal anesthesia, local anesthetics are removed from the subarachnoid space via absorption and diffusion across the dural and arachnoid membranes [4]. Higher degrees of spinal blockade result in a larger surface of absorption and diffusion, resulting in the elimination of a higher percentage of local anesthetic over time than lower levels, resulting in a shorter duration of anesthesia [5]. If we can restrict the dermatomal spread of the local anesthetic drug, the duration of the spinal block with similar drug dosages will be longer. The position of a patient immediately after the intrathecal injection of local anesthetic agents affects not just the spread but also the duration of the spinal blockade accordingly [6]. There has not been much research conducted to determine the effect of the reverse Trendelenburg position (head down) on the height of a subarachnoid block. Consequently, we sought to compare the reverse Trendelenburg position of 10° with the universal supine position in terms of block characteristics and hemodynamic parameters during lower limb orthopedic surgeries performed under spinal anesthesia.

## Materials and methods

We conducted a single-centric, prospective, single-blind randomized clinical trial in a tertiary care center in India’s Sub-Himalayan region. Before beginning the study, we obtained approval from the institutional ethics committee, and we strictly followed the Helsinki Declaration, 2000. The study population was patients undergoing knee or below knee surgeries under subarachnoid blocks in the supine position. Hemodynamically stable adult patients of either sex between the ages 20 and 65 years with the American Society of Anesthesiologists (ASA) physical status 1-2 were involved in the study. Patients with any known medical, neurological, neuromuscular, or psychiatric illness were excluded. Patients with contraindications to spinal anesthesia such as bleeding disorder, local infection, or allergy to local anesthetic agents were also excluded. Consecutive patients who met the aforementioned criteria were considered for recruitment, and those who agreed to participate were enrolled in the study. Using a computer-generated randomization sequence, 60 patients were enrolled and randomly assigned to one of the two groups. Allocation concealment was accomplished through the use of serially numbered opaque sealed envelopes. In the supine group (group S), patients were positioned in the supine position, and in the Trendelenburg group (group T), patients were positioned in a 10° reverse Trendelenburg position after administering spinal anesthesia. In both groups, an intravenous line was secured in the arm opposite to the operative site with an 18G cannula, and loading was conducted with 10 mL/kg of a lactated ringer. Five lead electrocardiography, oxyhemoglobin saturation, and noninvasive blood pressure monitoring (systolic, diastolic, and mean) were attached. All above-mentioned parameters (baseline) were recorded before giving subarachnoid block.

Under aseptic precautions, an epidural catheter was inserted in L2-L3 intervertebral space using 18G Tuohy’s needle in a sitting position. After a 5 cm long insertion into the epidural space, the catheter was secured. Subarachnoid space was pierced in L3-L4 intervertebral space using a midline lumbar approach and a 26G Quincke BD dura cutting spinal needle (0.45 mm × 90 mm). Heavy bupivacaine of 12.5 mg was slowly administered intrathecally at a rate of 0.2 mL/s. The spinal needle’s bevel end was kept pointing downward. Patients in group T were then turned supine with 10° tilt in the reverse Trendelenburg position, whereas those in group S remained supine throughout the procedure. Using standard mathematical calculations, a reversal of Trendelenburg’s position of 10° was achieved.

The onset of sensory block was measured from the time the drug was injected into the subarachnoid space until complete analgesia was achieved bilaterally at the level of T12 (anterior superior iliac spine). Sensory block was checked bilaterally in the midclavicular line using the pinprick method for pain, every 30 s until sensory block onset and then every 15 min until regression to two segments below maximum level. The maximum level of sensory block was defined as the level of block after 20 min. Two-segment regression time was calculated as the time it took for a block to be regressed to two segments below its maximum block level. The time to regress to T12 was defined as the duration of the sensory block. The duration of analgesia was defined as the requirement for the first analgesic dose. Motor block was evaluated every 30 s until the onset of the block and then every 15 min until the block was resolved (withheld during surgery). The time from intrathecal injection to modified Bromage score 3 was used to calculate the onset of motor block, and the time from modified Bromage score 3 to 0 was used to calculate the duration of motor block. If adverse effects such as hypotension or bradycardia occurred, they were observed, noted, and managed accordingly. A clinically significant hypotension episode was defined as a systolic blood pressure of less than 90 mmHg or mean blood pressure of less than 20% of baseline. The total number of doses of 6 mg ephedrine intravenous stat used to treat hypotension was calculated. Bradycardia was defined as a heart rate of fewer than 45 beats per minute and was treated with an intravenous injection of 0.6 mg atropine, which was repeated if necessary.

Statistical analysis

G*Power version 3.1.9.7 (Düsseldorf, Germany: The Heinrich Heine University Düsseldorf) was used to calculate the sample size. A sample size of 23 in each group was calculated on the basis of a 5% type 1 error and an 80% power of the study to detect a difference of one dermatomal segment. The data were presented in the form of frequency, percentage, mean, and/or standard deviation. To compare categorical variables, the chi-square test was used. To compare quantitative variables between the two groups, a t-test was used. A p-value of <0.05 was considered statistically significant.

## Results

**Figure 1:**
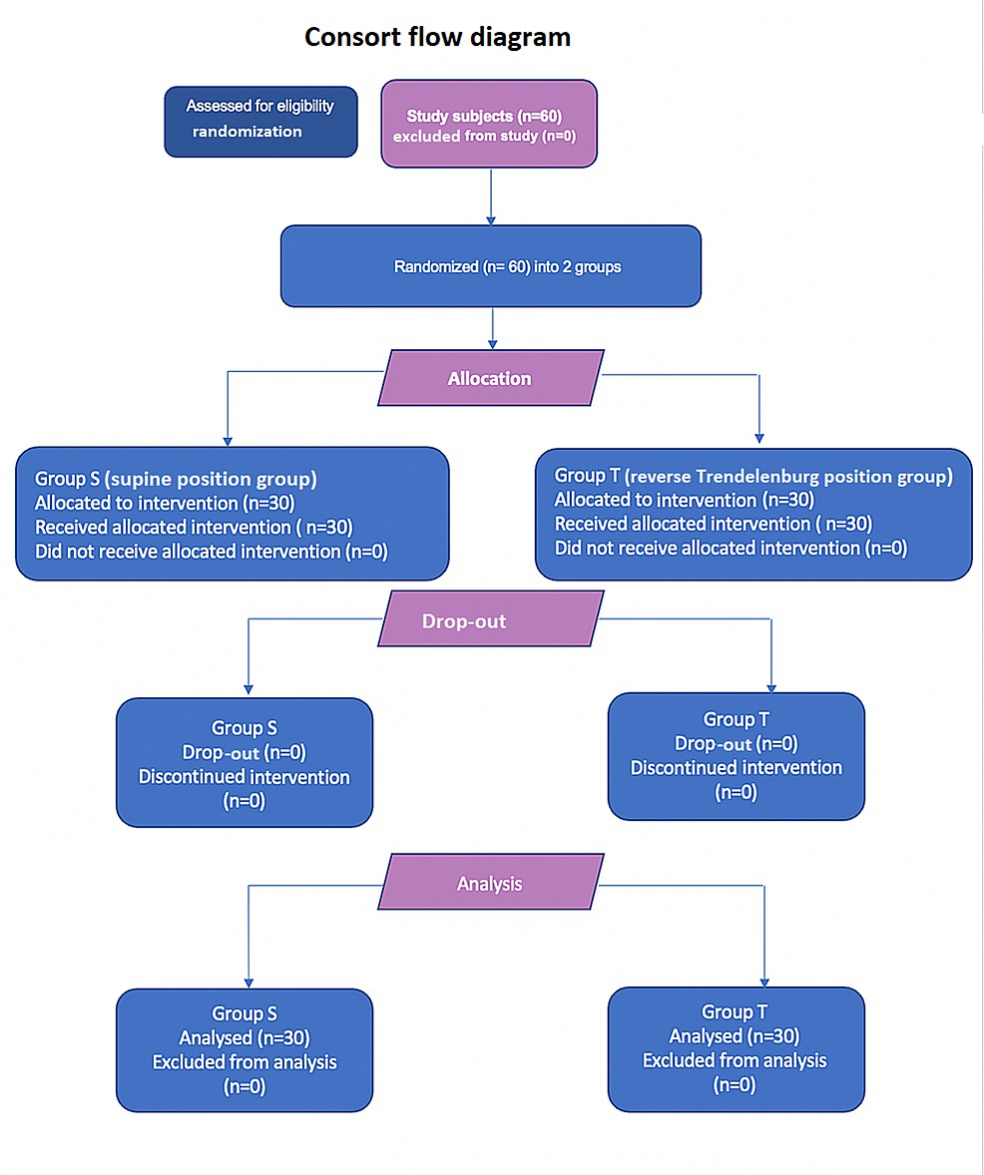
Flow chart of patients recruited and analyzed in two groups.

All 60 subjects enrolled were included in the analysis and there were no dropouts (Figure 1). Sociodemographic parameters of recruited patients including age, gender, height, weight, BMI, ASA grade, and duration of surgery were compared and summarized in Table 1.

**Table 1:** Sociodemographic and clinical determinants of recruited patients. BMI: body mass index; ASA: American Society of Anesthesiologists, N: number of patients

Variables	Supine position group (n=30) Mean ± SD or N	Reverse Trendelenburg position group (n=30) Mean ± SD or N
Age (years)	42.76±12.35	44.06±12.51
Sex (male:female), N	21:9	21:9
Height (cm)	160.56±6.53	158.86±3.52
Weight (kg)	59.20±5.92	58.43±5.31
BMI (kg/m^2^)	22.97±1.67	23.24±2.57
ASA grade (I: II), N	28:2	30:0
Duration of surgery (min)	77.10±16.50	76.63±19.17

**Figure 2:**
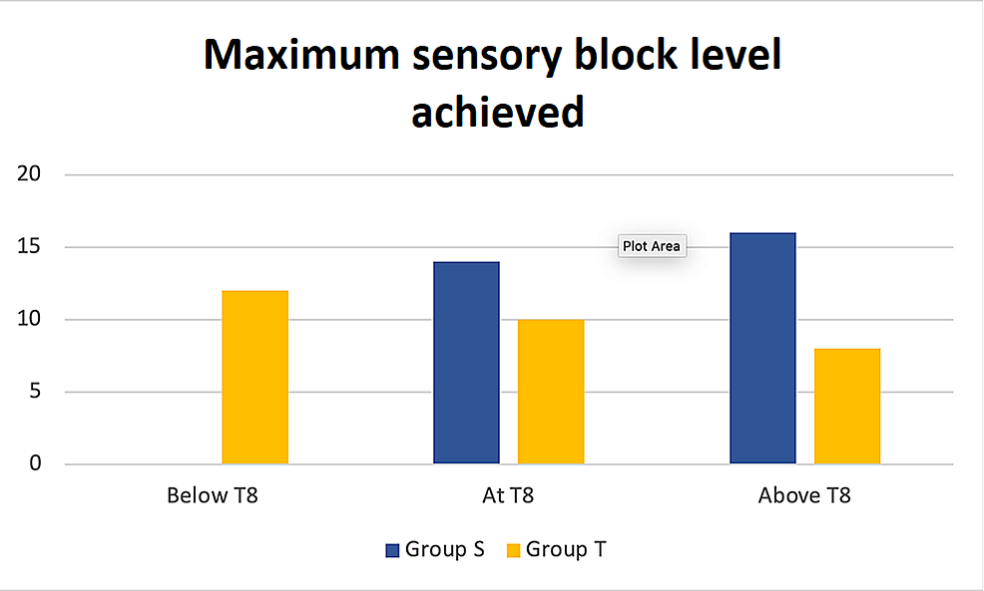
Maximum sensory block levels achieved: below T8, at T8, and above T8. Group S: supine position group; group T: reverse Trendelenburg position group

**Figure 3:**
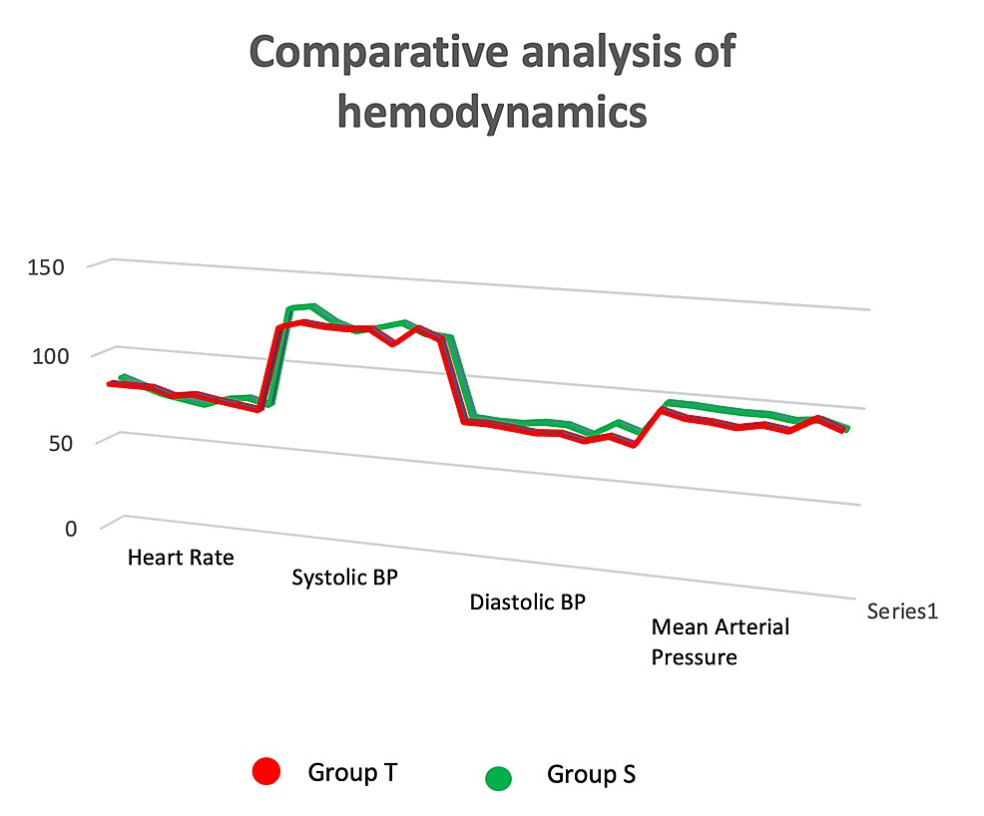
Line diagram showing comparative analysis of heart rate (HR), systolic blood pressure (SBP), diastolic blood pressure (DBP), and mean arterial pressure (MAP) among the two groups. Group S: supine position group; group T: reverse Trendelenburg position group

The onset of sensory block was significantly slower in the patients of group T compared to those of group S. Maximum sensory block level was significantly higher in group S than in group T. In group T, 26.6% had a sensory block level above T8, whereas in group S, 86.6% patients had a sensory block level above T8 (Figure 2). Duration of sensory and motor block and duration of analgesia were significantly higher in group T than in group S (Table 2). Heart rate, systolic, diastolic, and mean atrial pressure remained within physiological range to more extent in group T than in group S (Figure 3).

**Table 2:** Sensory block and motor block characteristics. N: number of patients

Variable	Supine position group (n=30) Mean ± SD or N	Reverse Trendelenburg position group (n=30) Mean ± SD or N	p-Value
Onset of sensory block (seconds)	127.33±47.55	150.60±39.77	0.044
Maximum sensory block level T4, T5, T6, T7, T8, T9, T10	0, 2, 12, 12, 4, 0, 0	0, 0, 3, 5, 10, 11, 1	<0.0001
Segment blocked above T12	5.10±1.06	3.96±0.99	<0.0001
Two segment regression time (minutes)	72.50±7.96	85.20±18.90	<0.01
Onset of motor block (seconds)	274.00±52.10	282.33±66.47	0.591
Duration of sensory block till T12 (minutes)	122.33±12.98	146.16±16.54	<0.01
Duration of motor block till T12 (minutes)	97.00±10.79	104.00±9.13	0.009
Duration of analgesia	165.36±6.83	180.46±8.19	<0.0001

Among adverse effects, bradycardia was not observed in any patient of both groups. The hypotension was observed in 33% (9/30) patients in group S, whereas none (0/30) of the patients in group T exhibited the hypotension.

## Discussion

According to the known literature and clinical experience with hyperbaric bupivacaine, Trendelenburg’s position has been extensively used for increasing the level of spinal anesthesia. Simultaneously, there is a paucity of literature utilizing the reverse Trendelenburg position, and even then, only in obstetric patients [7,8]. Given the possibility of insufficient spinal block height, anesthesiologists may not prefer a slight reverse Trendelenburg position in spinal anesthesia. The thoracic vertebral region has an upward slope in the reverse Trendelenburg position. In spinal anesthesia, it prevents cephalic shifting of hyperbaric local anesthetics to the upper thoracic nerve roots [8]. Increased spinal block can result in increased hypotension, bradycardia, decreased cardiac output, and decreased peripheral resistance [9]. The incidence of fall in blood pressure with a higher level of spinal block (T7 or above) can be as high as 60% [10]. The incidence of hypotension is higher for the elderly population because of pathological or exaggerated physiological changes in old age [11]. Hypotension can further trigger cardiac events and increase the incidence of postoperative mortality [12].

Lower spinal block levels are associated with a lesser incidence of hypotension and a decrease in blood pressure (mean arterial pressure {MAP}). This is because of the increase in sympathetic activity causing compensatory vasoconstriction in the region above the spinal anesthesia (thoracic region and upper torso) [13]. This increases the heart rate and stroke volume, which in turn increases cardiac output. An adequate level of spinal block for lower limb orthopedic surgery is T10. If the level of blockage is controlled to T10, it can avoid the side effects of high spinal block. Because of multiple factors affecting the level of spinal anesthesia, it is not always easy to control the level of spinal block [14].

The patient’s position may be critical in determining the final levels of motor and sensory blocks. Chang et al. previously studied the effect of spinal anesthesia in supine and prone position surgeries with isobaric bupivacaine and found no difference in sensory and motor block levels [15]. Toptaş et al. studied the effects of hyperbaric and isobaric bupivacaine on the spinal block. They discovered that hyperbaric bupivacaine causes significantly less heart rate variability and more stable hemodynamics [16]. In the present study, the mean time taken to achieve T12 sensory block was significantly delayed in group T as compared with that in group S. We have maintained a 10° reverse Trendelenburg tilt from the very beginning, and we have used hyperbaric drug. It may have led to a slower ascend of hyperbaric bupivacaine causing the slower onset of sensory block in group T. However, it may have no clinical relevance as delayed onset by 20-30 s is hardly going to influence our clinical goal. Nonetheless, this slower onset can explain more stable hemodynamic and comparatively low spinal block levels in group T.

The present study is supported by Poredos and Novak; they found that sensory anesthesia block was significantly higher in the high spinal group than in the low spinal group [17]. Infante et al. conducted a study and discovered that the maximum proximal spread of spinal block was significantly greater in the supine group than in the 30° torso elevation group [6]. The present study showed that the average number of segments blocked above T12 was significantly lower in group T than in group S. Thus, we conclude that giving 10˚ reverse Trendelenburg position in spinal anesthesia can help in controlling the level of sensory block. We also discovered that two-segment regression time was significantly slower in the group T when compared to group S. Therefore, we concluded that giving 10° reverse Trendelenburg position may prolong two-segment regression time in group T as compared with group S. One such study conducted by Lee et al. using head-up position showed similar results in cesarean section [8]. Results of our study are also supported by the study conducted by Infante et al., in which they reported that time taken for two-segment regression was significantly faster in the horizontal group than in the group with a torso elevation of 30° [6].

In the present study, we discovered that the sensory block duration was prolonged in group T as compared with group S. However, no study involving the use of reverse Trendelenburg position during spinal anesthesia for lower limb surgeries to compare the duration of sensory block was found in the literature. In our study, we discovered that motor block duration was also significantly prolonged in group T, thus showing that reverse Trendelenburg position may produce a longer duration of motor block. The present study is supported by a study conducted by Infante et al., who concluded that complete motor recovery time was significantly shorter in the horizontal group [6].

In the present study, it was also discovered that the total duration of analgesia was significantly longer in group T than in group S. This study is supported by the study conducted by Infante et al., in which they reported that pain appeared significantly faster in the horizontal group than in the group with torso elevation of 30° [6]. The overall incidence of hypotension in the study conducted by Poredos and Novak was 35% in the high spinal group and 10% in the low spinal group [17]. Those results were consistent with the results of the present study. This study also reported that clinically relevant hypotension occurred in only 33% of patients in group S and none in group T. Our study hypothesize that the reverse Trendelenburg position can be used as a strategy for reducing spinal anesthesia-induced hypotension and other hemodynamic deterioration and minimizing the peak block level to as low as possible for the planned procedure. There have been very few studies in which reverse Trendelenburg position has been used for controlling the height of spinal block using hyperbaric drugs.

## Conclusions

We conclude that keeping patients in a 10° reverse Trendelenburg position after spinal anesthesia can significantly control the height of the sensory block, resulting in stable hemodynamics. More large-scale clinical trials with larger sample sizes are needed to validate our hypothesis.

Controlling the level of spinal anaesthesia is easier said than done. The present study concludes that maintaining the patients in 10˚ reverse Trendelenburg position after giving spinal anaesthesia can significantly limit the height of the sensory block, which provides stable hemodynamics. Controlling the height of the block is also associated with an increase in the duration of sensory block and analgesia. Our findings can be of paramount importance, especially in geriatric and high-risk patients where even a small insult in the form of hypotension or bradycardia can trigger life-threatening events. Larger clinical trials are required to validate our hypothesis.
